# Patient satisfaction and survey response in 717 hospital surveys in Switzerland: a cross-sectional study

**DOI:** 10.1186/s12913-020-5012-2

**Published:** 2020-03-02

**Authors:** Thomas V. Perneger, Isabelle Peytremann-Bridevaux, Christophe Combescure

**Affiliations:** 1 Division of clinical epidemiology, Geneva University Hospitals, and Faculty of medicine, University of Geneva, Geneva, Switzerland; 2 Center for primary care and public health (Unisanté), University of Lausanne, Lausanne, Switzerland

**Keywords:** Patient satisfaction, Survey response, Non-response bias

## Abstract

**Background:**

The association between patient satisfaction and survey response is only partly understood. In this study, we describe the association between average satisfaction and survey response rate across hospital surveys, and model the association between satisfaction and propensity to respond for individual patients.

**Methods:**

Secondary analysis of patient responses (166′014 respondents) and of average satisfaction scores and response rates obtained in 717 annual patient satisfaction surveys conducted between 2011 and 2015 at 164 Swiss hospitals. The satisfaction score was the average of 5 items scored between 0 and 10. The association between satisfaction and response propensity in individuals was modeled as the function that predicted best the observed response rates across surveys.

**Results:**

Among the 717 surveys, response rates ranged from 16.1 to 80.0% (pooled average 49.8%), and average satisfaction scores ranged from 8.36 to 9.79 (pooled mean 9.15). At the survey level, the mean satisfaction score and response rate were correlated (r = 0.61). This correlation held for all subgroups of surveys, except for the 5 large university hospitals. The estimated individual response propensity function was “J-shaped”: the probability of responding was lowest (around 20%) for satisfaction scores between 3 and 7, increased sharply to about 70% for those maximally satisfied, and increased slightly for the least satisfied. Average satisfaction scores projected for 100% participation were lower than observed average scores.

**Conclusions:**

The most satisfied patients were the most likely to participate in a post-hospitalization satisfaction survey. This tendency produces an upward bias in observed satisfaction scores, and a positive correlation between average satisfaction and response rate across surveys.

## Background

Patient satisfaction ratings are routinely used to assess the quality of hospital care [1–4]. Ideally, to obtain an unbiased assessment, one would measure the satisfaction of every patient in a representative sample. In real life, however, participation rates in satisfaction surveys are commonly below 50% [5, 6], and may be decreasing over time; e.g., in the UK, participation rates in the Adult Inpatient Survey went from 59% in 2005 to 47% in 2014 [7]. Whether non-response biases survey results depends on the relationship between a patient’s satisfaction and her or his propensity to participate in the survey [8, 9]. Current evidence suggests that potential for bias exists. Firstly, subsets of patients who report low satisfaction levels are also less likely to participate in a survey: younger patients and the very old, those who require longer hospital stays, and psychiatry patients [9]. Secondly, patients who return a satisfaction survey only after reminders report lower satisfaction scores than those who respond immediately [9]. Finally, two studies have shown that response rates and average satisfaction levels are positively correlated at the hospital or provider level. Among cancer patients treated at 158 hospitals in the UK, survey item averages and response rates had Spearman correlation coefficients between 0.03 and 0.44 [10]. Similarly, across 80 primary care practices in Massachussetts, the correlation between average outpatient satisfaction and survey response rate was 0.52 [11].

The simplest explanation for the association between average satisfaction and response rates is causality: a more satisfied patient may be more likely to return the survey. Thus if patients were on average more satisfied in hospital A than in hospital B, then mean satisfaction scores would be higher in hospital A than in hospital B, and at the same time the survey response rate would also be higher in hospital A than in hospital B. Of note, since satisfied patients are over-represented among respondents in this scenario, all observed mean satisfaction scores are biased upward. The association between a patient’s satisfaction and her or his probability of participating in the survey plays an essential role in these phenomena, yet little is known about its shape.

In this study, we used the results of annual satisfaction surveys conducted by Swiss hospitals between 2011 and 2015 to examine the relationship between satisfaction and response. We aimed to a) verify if the response rate and average satisfaction were indeed correlated across hospitals, and estimate the magnitude of this correlation, and b) estimate the shape of the response propensity function.

## Methods

### Study design and setting

Cross-sectional analysis of hospital satisfaction surveys. Both individual patient responses and survey averages were analyzed. We used data transmitted by participating hospitals to the Swiss National Association for the Development of Quality in Hospitals and Clinics (ANQ, www.anq.ch), an association established in 2009 for the purpose of measuring quality indicators and supporting quality improvement in Swiss hospitals. ANQ collects quality indicators in areas of acute care, psychiatry and rehabilitation, and patient satisfaction is one of the quality indicators for acute care hospitals. ANQ satisfaction surveys started in 2011 and used the same five-item questionnaire until 2015; from 2016 on the questionnaire was modified. The survey procedures, including the questionnaire layout and the content of the cover letter, were prescribed in a reference manual. Hospitals identify all patients 18 years old or older who are discharged from the hospital during a 1 month period, but the surveys are conducted by independent entities. Five survey providers (4 private and one public) were approved by the ANQ during the study period. Only one mailing was performed, without reminders. A team based at a university institute was in charge of analyzing the data each year and of producing an annual report. Survey results are publicly available on the website of the ANQ, with hospital identifiers. For this study, the authors submitted a research proposal to ANQ and obtained a dataset for the 5-year period of 2011–2015, without any patient or hospital identifiers. Because this was a secondary analysis of publicly available data, approval by a research ethics committee was not sought. Furthermore, in Switzerland, patient satisfaction surveys are considered to be hospital management activities and do not require approval by a research ethics committee.

### Study variables

Patient-level variables were age, gender, insurance status (basic insurance or private insurance), and ratings of the 5 satisfaction items (Additional file 1). Satisfaction items were also averaged over each survey. Additional variables at the survey level were the sample size of each survey, the response rate, the hospital classification code (6 categories: university hospital, general hospital in 4 categories by size, specialized clinics), and language area (German, French, Italian).

### Statistical analysis

The sample size was not pre-specified; we used all available data. We described the distributions of the hospital surveys and of the respondents by year, hospital type, language area, and for patients only, insurance status, age and sex. We obtained the distributions of the 5 survey items in the whole sample, and as the item scores were highly correlated at the individual level (Cronbach alpha 0.96), we computed an individual summary score as the average of the 5 items. We computed the average score for each item and for the summary score in each survey.

We described the survey response rates by year, hospital type, and language area, weighting each observation by the number of questionnaires mailed out (weights standardized to sum to the total number of surveys). To test differences between subgroups we used mixed linear models, with a random intercept defined by any given hospital. We did the same analysis for the satisfaction score means, but this time weighting the observations by the number of respondents (weights were standardized to sum to the total number of surveys). To examine the association between response rates and satisfaction means, we computed Pearson correlation coefficients, overall and in strata defined by calendar year, language area, hospital type, and insurance status of the majority of respondents. We also obtained a scatter-plot of response rate by mean satisfaction, with dot size proportional to the number of respondents.

### Modeling the association between satisfaction and survey response

We used both individual satisfaction data and survey averages (mean satisfaction and response rate) to model the probability that a patient will participate in the survey as a function of her or his satisfaction (Additional file 2). Of note, we were not able to identify multiple hospital stays for the same patient. The logit of the probability of response was represented by a piecewise linear spline function with up to 5 nodes; this type of function does not impose any particular shape to the relationship, and is only constrained by the positions of the nodes. Once the function coefficients were defined (see below), the function was applied to the distribution of satisfaction scores among respondents in each survey, and used to derive the distribution of satisfaction scores in all patients who were invited to participate (e.g., if there were 4 respondents with a satisfaction score of 8, and the function stipulated a response rate of 0.4 at that level, the estimated total number of patients with a satisfaction score of 8 was 10). The regression coefficients that characterize the function were selected so as to minimize the sum of squared deviations between the estimated numbers of invited patients and the actual numbers, across all surveys; in other words we selected the function that came closest to reproducing the observed response rates. The optimization was solved using an iterative numerical procedure (using the R package *nlm*). Of note, a key assumption of this procedure was that the same response function applied to all surveys. To check the robustness of the procedure, we used 3 sets of initial parameter values and 2 sets of node positions. We represented the response function graphically. To estimate the magnitude of non-participation bias, we plotted the (inferred) full-sample satisfaction means as a function of measured satisfaction means among respondents, across all surveys.

The analyses were conducted in SPSS version 24 and Stata version 15, and the function estimation was programmed in R.

## Results

We obtained data for 171′141 non-empty survey responses from 936 annual hospital surveys, and excluded 219 surveys that included fewer than 50 responses each (total 5127 responses). The analysis shown hereafter includes the remaining 717 hospital surveys and 166′014 patient responses. Surveys were conducted at 164 different hospitals: 111 provided data from 5 annual surveys, 30 from 4 surveys, 5 from 3 surveys, 9 from 2 surveys, and 9 provided data from 1 survey only. Annual numbers of hospitals ranged from 130 to 149, and about 30′000 patients participated each year (Table 1). The majority of hospitals and patients were from the German-speaking area of the country. Most hospitals were general hospitals of medium size, and most served predominantly patients covered by basic compulsory health insurance. Among the respondents, one third had supplemental private insurance, over half were women, and a majority was older than 60 years.

**Table 1:** Characteristics of hospitals and survey respondents

	Hospitals, N (%) (*N* = 164)	Respondents, % (*N* = 166′014)
Year		
2011	130 (79.3)	19.5%
2012	143 (87.2)	19.8%
2013	146 (89.0)	20.3%
2014	149 (90.9)	19.9%
2015	149 (90.9)	20.5%
Language		
German	120 (73.2)	75.0%
French	34 (20.7)	20.1%
Italian	10 (6.1)	4.9%
Hospital type		
University hospital (level 1)	5 (3.0)	13.7%
General hospital (level 2)	44 (26.8)	38.2%
General hospital (level 3)	30 (18.3)	18.4%
General hospital (level 4)	42 (25.6)	17.9%
General hospital (level 5)	18 (11.0)	5.0%
Specialized clinics	25 (15.2)	6.9%
Insurance status	(^a^)	
Public insurance	135 (87.7)	66.7%
Private insurance	19 (12.3)	33.3%
Gender		
Women		55.2%
Men		44.8%
Age, years		
18–39		19.7%
40–59		25.1%
60–79		39.9%
80–110		15.0%

^a^Insurance status of majority of patients

### Response rates

In the course of the 717 retained surveys, 350′972 patients were contacted and 166′014 responded, for an overall response rate of 47.3%. Each survey contacted between 70 and 2794 patients (mean 489.5, quartiles 211, 346, 584) and obtained 50 to 1067 responses (mean 231.5, quartiles 103, 163, 282). The 717 survey response rates ranged from 16.1 to 80.3% (unweighted mean 49.8%, quartiles 43.3, 49.2, 56.2%). Applying hospital weights proportional to the average number of patients contacted over the years, the correlations of response rates between consecutive years were 0.78 (2011–12), 0.68 (2012–13), 0.69 (2013–14) and 0.78 (2014–15).

The mean weighted survey response rates decreased slightly over time (Table 2, first column). The rates were lower in the Italian-speaking hospitals than in other linguistic regions. They were lowest in university hospitals, and highest in specialized clinics, and also lower in hospitals that treated a majority of patients with basic insurance than in hospitals that treated a majority of patients with supplemental private insurance.

**Table 2:** Survey response rates, mean satisfaction scores, and Pearson correlations between response rate and mean satisfaction for 717 surveys in 164 Swiss hospitals, 2011–2015

	Response rate^a^ (%)		Mean satisfaction^b^		Pearson r^b^
	Mean (SD)	*P* value	Mean (SD)	*P* value	
Overall	47.3 (8.8)		9.14 (0.24)		0.61
Year:		0.001		0.001	
2011	48.9 (8.6)		9.15 (0.25)		0.72
2012	47.9 (9.7)		9.16 (0.21)		0.66
2013	48.3 (9.3)		9.14 (0.24)		0.59
2014	45.6 (8.2)		9.12 (0.25)		0.56
2015	46.1 (7.6)		9.15 (0.23)		0.57
Language:		0.049		< 0.001	
German	47.7 (9.1)		9.19 (0.20)		0.67
French	47.0 (8.0)		8.99 (0.30)		0.62
Italian	42.8 (6.4)		9.11 (0.21)		0.64
Hospital type:		< 0.001		< 0.001	
University hospital	40.0 (6.1)		8.92 (0.17)		−0.06
Large general (level 2–3)	47.1 (7.7)		9.11 (0.21)		0.53
Small general (level 4–5)	50.3 (8.5)		9.27 (0.20)		0.54
Specialized clinic	69.3 (9.0)		9.41 (0.22)		0.53
Insurance status (majority of patients)		< 0.001		0.008	
Basic insurance	46.2 (8.3)		9.11 (0.23)		0.56
Private insurance	56.8 (7.4)		9.39 (0.12)		0.34

^a^Weighted by sample size of patients contacted for survey

^b^Weighted by sample size of respondents in survey

### Satisfaction ratings

The distributions of responses to the 5 questions were asymmetric, with more than half of the respondents at the maximum score of 10 for each item (Table 3). At the individual level, between-item Pearson correlation coefficients ranged from 0.51 to 0.78, and the internal consistency coefficient (alpha) was 0.88. A total score computed as the average of the 5 items had a mean of 9.15 (SD 1.28).

**Table 3:** Distributions of 5 survey items, in percent, among survey respondents

	Would return to this hospital	Rating of quality of care	Received understandable answers from doctors	Received understandable answers from nurses	Treated with respect and dignity
0 (lowest)	1.1	0.4	0.3	0.2	0.3
1	0.3	0.2	0.3	0.2	0.2
2	0.5	0.4	0.5	0.4	0.4
3	0.6	0.5	0.7	0.6	0.4
4	0.5	0.6	0.7	0.7	0.5
5	2.1	1.7	1.7	1.9	1.2
6	1.4	1.7	1.9	2.2	1.1
7	3.3	4.4	4.2	5.0	2.4
8	11.4	16.6	12.4	15.0	7.9
9	11.3	19.6	15.0	17.2	13.6
10 (highest)	67.4	53.8	55.5	51.4	72.0
Did not ask any questions	–	–	6.8	5.1	–
Mean (SD)	9.15 (1.73)	9.02 (1.53)	9.13 (1.55)^a^	9.03 (1.53)^a^	9.39 (1.36)

^a^The response “Did not ask any questions” was treated as a 10

We obtained average scores for each of the 717 hospital surveys. The 5 item-specific survey means were highly correlated (Pearson correlation coefficients between 0.80 and 0.94). At the survey level, the internal consistency of the 5 item means was 0.96. For the total score, survey means ranged from 8.36 to 9.79, and the average, weighted proportionally to the number of respondents in each survey, was 9.14 (SD 0.24, quartiles 8.98, 9.13, 9.33). Correlations of weighted total score averages between consecutive years were 0.80 (2011–12), 0.76 (2012–13), 0.82 (2013–14) and 0.86 (2014–15).

Average satisfaction scores remained stable over the 5 years (Table 2, middle column). They were lower in French-speaking hospitals than in other language areas, and lower in large hospitals than in small hospitals and in specialized clinics. Satisfaction scores were also higher in hospitals that treated a majority of privately insured patients. These associations persisted in a multivariable regression model (not shown).

### Association between mean satisfaction scores and response rates

**Fig. 1:**
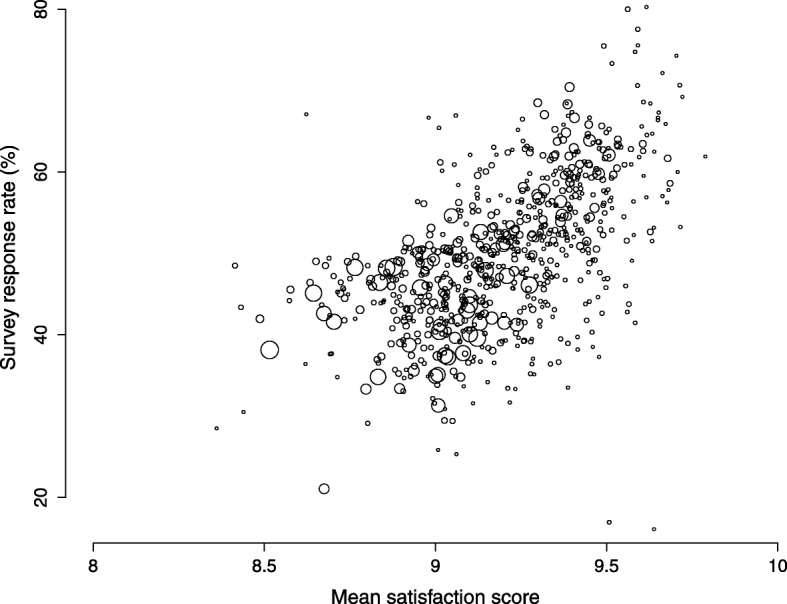
Scatter-plot of observed survey response rate by mean satisfaction score, in 717 hospital satisfaction surveys, Switzerland, 2011–2015. Circle size reflects sample size

Globally, higher mean satisfaction scores were associated with higher survey response rates (Fig. 1). The frequency-weighted Pearson correlation coefficient was 0.61. The association was seen in each calendar year, but it was highest in 2011 and decreased thereafter, reaching 0.57 in 2015 (Table 2, last column). The correlation was similar in the three language areas. It was notably absent among the 5 university hospitals (totalling 25 surveys), but similar for other hospital types. It was also lower among hospitals serving a majority of patients with private insurance.

### Estimation of the probability of responding for individuals

**Fig. 2:**
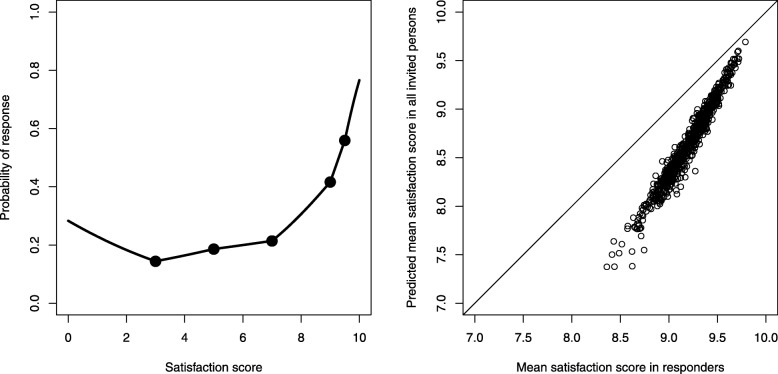
Left: Relationship between an individual’s satisfaction score and probability of response. The dots represent nodes where the slope is allowed to change (satisfaction scores of 3, 5, 7, 9, 9.5). Right: Plot of the mean statisfaction score observed in responders and predicted by the model among all invited persons (responders and non responders). Each survey is represented by a circle. The diagonal line is the identity line, and the vertical distance between a circle and the identity line corresponds to non-response bias

The estimation procedure that used 5 nodes for the spline function (located at satisfaction scores of 3, 5, 7, 9, and 9.5) resulted in a “J-shaped” function (Fig. 2, left panel): the probability of response was low, around 20%, for satisfaction scores between 3 and 7; it increased sharply to about 70% for those maximally satisfied, and increased only slightly for the least satisfied. Of note, the model converged to the same coefficients regardless of starting values, and an alternative model with four nodes (at 4, 6, 8 and 9) produced a somewhat more irregular but globally similar shape (Additional file 2). We used the response function to infer satisfaction scores in non-respondents and computed the hypothetical true satisfaction average that would have bee obtained if all contacted patients had participated. The resulting mean scores were always lower than the observed mean scores, and the amount of bias was inversely associated with the survey mean (Fig. 2, right panel). The observed and imputed mean scores were highly correlated (Pearson correlation coefficient 0.98).

## Discussion

This analysis of 717 annual patient surveys conducted at 164 Swiss hospitals between 2011 and 2015 revealed a positive correlation (r = 0.61) between the average satisfaction score and the survey response rate. This correlation was stronger than previously reported [10, 11]. It was present in each calendar year, in all three language areas, and for all hospital types excepting the subset of 5 large university hospitals.

An original contribution of this study is the estimation of the shape of the individual response propensity function. For an individual, the estimated probability of participating in the survey increased sharply when satisfaction was high, above 7 or 8, but remained approximately flat across middle satisfaction levels, and increased slightly among the least satisfied. In retrospect, this “J-shape” seems compatible with an intuitive understanding of human motivations – the happy are grateful and eager to please, the unhappy need to vent, and those in-between remain (mostly) silent. The sharp up-turn in the probability of response at high satisfaction scores explains why the association between response rates and mean satisfaction scores was so strong across surveys: a relatively small shift of the distribution to higher satisfaction levels would cause a notable increase in response rates.

Globally, non-participation caused an optimistic bias for all hospitals, but this bias was strongest for the hospitals that achieved the lowest satisfaction results. Thus non-participation bias compressed the differences between the best and worst hospitals; this may induce complacency among the poorest performers. Nevertheless, observed and response-corrected averages were highly correlated, which suggests that non-response would not distort the rankings of hospitals, at least if the same survey methods are used.

We believe that the observed association between satisfaction and propensity to return the satisfaction questionnaire may be causal, mediated by a sense of gratitude toward the hospital that provided excellent care, and to a lesser extent by a sense of annoyance at a hospital that provided poor care. However, causality cannot be established by an observational study, and confounding and bias should also be considered. One candidate confounder is the patient’s inherent desire to please, or agreeableness [12]. Patients who are eager to please others might both rate the care received highly, and agree to fill in the questionnaire and send it back; conversely those who care less about pleasing others may be both more severe in their assessments and less likely to participate in the survey. The tendency to rate care highly can be measured reliably [13], but whether it correlates with participation is not known. For this characteristic to explain our findings, hospitals would need to vary in their patient mix with regard to rating tendency, which seems unlikely.

Another patient-level confounder may be a mainstream versus marginal social status. A typical Swiss hospital is not primarily designed to care for socially marginalized patients, such as undocumented migrants, or patients with psychiatric disorders, drug habits, or non-standard gender identity. Even if marginalized patients were treated in the same way as mainstream patients – which is not always the case [14] – this may not be optimal for their particular situation or preferences. Furthermore, these social groups may also be more difficult to reach by means of mailed surveys [15].

Finally, let us consider bias. Selection bias at the hospital level is not a convincing explanation; it would require that hospitals with low satisfaction scores but high response rates, and vice-versa, be less likely to participate in the national quality indicator measurements. We see no plausible mechanism for this. Measurement bias may have occurred through the use of three different language versions, but this would not bias the correlation between mean satisfaction scores and response rates within language areas.

A strong feature of this study is the large sample size, both in terms of patients and of surveys. The availability of person-level and survey-level data allowed an estimation of the shape of the response propensity function; we are not aware of similar estimations done previously. A key weakness was the lack of information about non-responders, beyond their number; such information would have allowed a more precise analysis of the propensity to respond. Survey response is determined by many factors other than the level of satisfaction, which we were not able to explore. Finally, our approach to estimating the shape of the satisfaction-response function required the assumption that the same function applied to all surveys, but we were not able to verify this assumption. Future research should verify if the J-shaped function we described applies in other contexts. If indeed the same survey response function was generally applicable, non-response would not bias comparisons between providers.

## Conclusion

This pooled analysis of 717 hospital surveys and 166′014 patient responses suggests that the most satisfied patients are the most likely to return a hospital satisfaction questionnaire; to a lesser extent, the least satisfied patients are also more likely to participate than those in the middle. This individual response pattern produced an association between average satisfaction and response rate at the hospital level. Furthermore, this phenomenon caused an optimistic bias, whereby mean satisfaction scores appeared higher than they should be. While this bias affected all results, it was most pronounced for hospital surveys with the lowest satisfaction scores.

## Supplementary information

**Additional file 1.** Questions asked in the surveys, in the original languages, and translated.

**Additional file 2.** Estimation of the satisfaction-response function.

## Data Availability

Data are not available from the authors. Results of the surveys are publicly available on the website of the Swiss National Association for the Development of Quality in Hospitals and Clinics (ANQ, www.anq.ch). A database of individual survey responses was provided to the authors by the ANQ for the sole purpose of estimating the satisfaction-response function; access to this dataset can be requested from the ANQ.

## Abbreviation

ANQ: Swiss National Association for the Development of Quality in Hospitals and Clinics
