# Supplementary material for: Patient satisfaction and survey response in 717 hospital surveys in Switzerland: a cross-sectional study
Source: BMC Health Serv Res. 2020 Mar 2;20:158. doi: 10.1186/s12913-020-5012-2 (PMC7052977; doi:10.1186/s12913-020-5012-2)
Supplement: Supplementary file 1 — Additional file 1. Questions asked in the surveys, in the original languages, and translated. [file 12913_2020_5012_MOESM1_ESM.docx]

Additional file 1. Questions asked in the surveys, in the original languages, and translated

| Authors‘ translation from French | German | French | Italian |
| --- | --- | --- | --- |
| Would you choose to return to our hospital for similar care? | Würden Sie für dieselbe Behandlung wieder in dieses Spital kommen? | Choisiriez-vous de revenir dans notre hôpital pour une prise en charge similaire ? | Ritornerebbe nel nostro ospedale per lo stesso tipo di trattamento? |
| What do you think of the quality of the care you have received in the hospital | Wie beurteilen Sie die Qualität der Behandlung, die Sie erhalten haben? | Que pensez-vous de la qualité des soins que vous avez reçus à l’hôpital ? | Come valuta la qualità delle cure ricevute? |
| When you asked questions of doctors, did you receive understandable answers? | Wenn Sie Fragen an eine Ärztin oder Arzt stellten, bekamen Sie verständliche Antworten? | Lorsque vous avez posé des questions aux médecins, avez-vous reçu des réponses compréhensibles ? | Quando ha rivolto domande ad un medico, ha ottenuto risposte comprensibili? |
| When you asked questions of nurses, did you receive understandable answers? | Wenn Sie Fragen an das Pflegepersonal stellten, bekamen Sie verständliche Antworten? | Lorsque vous avez posé des questions aux infirmier(ère)s, avez-vous reçu des réponses compréhensibles ? | Quando ha rivolto domande ad una infermiera o ad un infermiere, ha ottenuto risposte comprensibili? |
| Do you think that hospital staff treated you with respect and preserved your dignity? | Wurden Sie während des Spitalaufenthaltes mit Respekt und Würde behandelt? | Estimez-vous que le personnel hospitalier vous a traité(e) avec respect et a préservé votre dignité ? | Durante la sua degenza è stata/o trattata/o con rispetto e la sua dignità è stata preservata? |
