# Supplementary material for: Patient satisfaction and survey response in 717 hospital surveys in Switzerland: a cross-sectional study
Source: BMC Health Serv Res. 2020 Mar 2;20:158. doi: 10.1186/s12913-020-5012-2 (PMC7052977; doi:10.1186/s12913-020-5012-2)

**Additional file 2: Estimation of the satisfaction-response function**

Patient satisfaction scores are observed only in persons who return the questionnaire. If the probability of response depended on the patient’s satisfaction, the observed satisfaction results would be biased. We aimed to estimate the shape of the function that links a patient’s satisfaction to his or her probability of participating in the survey. We modelled this relationship by a fully flexible spline function. To estimate the shape of the function (captured by its coefficients), we used the aggregated results (average satisfaction scores and survey response rates) of a large number of surveys (N=717); the selected coefficient estimates were those that predicted best the observed results. Then, for each survey, we extrapolated the distribution of the satisfaction scores in all invited persons (responders and non responders), and obtained the average score that would have been obtained with perfect participation.

Methods :

The first step was to model the relationship between the probability of response and the satisfaction score. To allow flexibility in the shape of the relationship, we modelled the logit of the probability of response by a piecewise linear spline function of the satisfaction score :

$logit P\left[ R=1 | S=s \right]= \alpha_{0}+\alpha_{1}s+\sum_{k=2}^{K} \alpha_{k}\left( s-s_{k-1} \right) 1_{s\geq s_{k-1}}$ (B1)

where *R* is the response variable (0 for non response and 1 for response), *S* is the satisfaction score, *s*_k_ are the a priori selected cut-offs of the score at which the slope of the logit can change and α_k_ are the slope parameters of the model to be assessed. With this model, the logit of the probability is α_0_ when the score is 0. For a score between 0 and *s*_1_, the logit of the probability is α_0_+ α_1_*s*, a linear function with slope α_1_. Between *s*_1_ and *s*_2_, the logit of the probability is α_0_+ α_1_*s* + α_2_(*s*-*s*_1_) = α_0_+ α_1_(*s*_1_*+s-s*_1_) + α_2_(*s*-*s*_1_) = (α_0_+ α_1_*s*_1_) + (α_1_+α_2_)(*s*-*s*_1_) : the logit is linear with a slope α_1_+α_2_. The parameter α_2_is the change in the slope between the intervals of the score (0; *s*_1_) and (*s*_1_; *s*_2_). As a generalization, the parameter α_k_ is the change in the slope between the intervals of the score (*s*_k-2_; *s*_k-1_) and (*s*_k-1_; *s*_k_). We assumed that the relationship between the probability of response and the level of satisfaction is identical across surveys.

The second step was to estimate the parameters α_k_. Our approach was as follows: the expected number of invited persons with a satisfaction score of *s* (responders + non responders) in each hospital and at each year was calculated from the number of responders with a satisfaction score of *s* and from the probability of response given in Equation (B1). The final estimated parameter values α_k_ were values that minimized the differences between the expected and actual numbers of invited persons in each survey. In other words, the parameters were set so as to best replicate the observed response rates across the 717 surveys.

In detail, the procedure was:

1. Initial values of the parameters α_k_ were assumed. (We used 3 different sets of initial parameter values: a constant propensity to participate independent of satisfaction level, increasing participation with satisfaction, and decreasing participation with satisfaction. All converged to the same final estimates.).
2. the observed number of responders in the hospital *i* at year *j* who reported a satisfaction score of *s* was denoted by $N_{s, responder}^{i,j}$. The number of invited persons (responders + non responders) in the hospital *i* at year *j* who have a satisfaction score of *s* was unknown since the score was not observed in non responders. However, its expectation $E_{s}^{i,j}$ was estimated by $N_{s, responder}^{i,j}/P[R=1|S=s]$ , where the denominator was obtained from Equation (A1). For instance, if 4 responders reported a score of 8 and $P[R=1|S=8]$=0.4, then we expected that 10 invited persons actually had a score of 8.
3. the overall expected number of invited persons assuming Equation (B1), denoted by $E^{i,j}$, was calculated as the sum over all scores of $E_{s}^{i,j}$
4. we defined a loss function capturing the difference between the actual numbers of invited persons in the hospital *i* at year *j* (${i.e., N}^{i,j}$) and the expected number $E^{i,j}$ :

$$L=\sum_{i,j} {[\ln\left( N^{i,j} \right)-\ln\left( E^{i,j} \right)]}^{2}$$

5. Steps 2) to 4) were iterated to minimize the loss function defined in step 4). We used the function *nlm* in R software with a convergence criterion (difference in coefficients between two successive iterations) of 10^-8^.
6. Finally, the mean statisfaction score was obtained for each survey from the expected numbers $E_{s}^{i,j}$ calculated with the estimates of the parameters α_k_.
7. To verify the robustness of the estimated function, we conducted this optimization using 2 sets of cutoff values for the spline function:

- first set: *s*_1_=4, *s*_2_=6, *s*_3_=8 and *s*_4_=9
- second set : *s*_1_=3, *s*_2_=5, *s*_3_=7, *s*_4_=9 and *s*_5_=9.5

Results :

The response function was “J-shaped”, both on the logit scale and on the probability scale, for both models (Figure B1). The estimated parameter values and the probabilites of responding to the survey at a given satisfaction level are given in Table B1. The correlations between estimated satisfaction means scores by the 2 models and the observed mean scores across the 717 surveys are shown in Figure B2.

Table B1. Parameter values and estimated probabilities of survey response according to patient satisfaction, for two models (Model 1: spline cutoff values at *s*_1_=4, *s*_2_=6, *s*_3_=8 and *s*_4_=9; Model 2: cutoff values at *s*_1_=3, *s*_2_=5, *s*_3_=7, *s*_4_=9 and *s*_5_=9.5)

|  | Model 1 | Model 2 |
| --- | --- | --- |
| Estimates of parameters |  |  |
| α_0_ | -1.0115 | -0.9280 |
| α_1_ | -0.1902 | -0.2836 |
| α_2_ | 0.5587 | 0.4361 |
| α_3_ | -0.4792 | -0.0652 |
| α_4_ | 1.3075 | 0.3939 |
| α_5_ | -0.0997 | 0.6695 |
| α_6_ |  | 0.7426 |
| Probability of response for a score of |  |  |
| 0 | 0.267 | 0.283 |
| 1 | 0.231 | 0.229 |
| 2 | 0.199 | 0.183 |
| 3 | 0.170 | 0.144 |
| 4 | 0.145 | 0.164 |
| 5 | 0.197 | 0.186 |
| 6 | 0.262 | 0.200 |
| 7 | 0.241 | 0.214 |
| 8 | 0.222 | 0.306 |
| 9 | 0.485 | 0.417 |
| 10 | 0.738 | 0.766 |

Figure B1 : Relationship between the satisfaction score and the logit of the probability of response (left panel) and the probability of response (rigth panel). The grey lines represent the results of the model with 4 cut-offs (Model 1) and the black lines the results of the model with 5 cut-offs (Model 2). The circles represent the cut-offs of score with a potential break in the relationship.


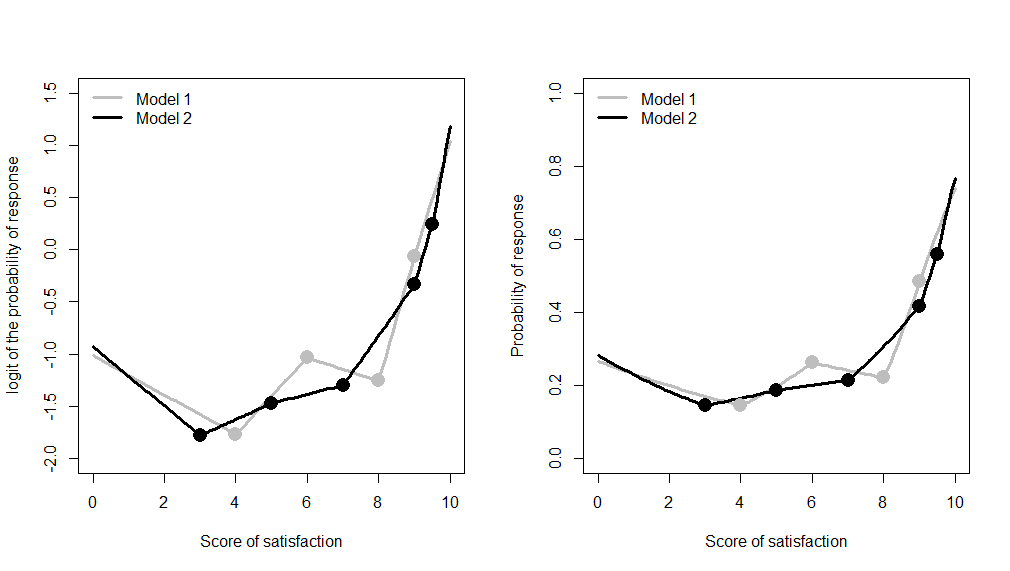


Figure B2 : Plot of the mean statisfaction score in responders and in all invited persons (responders + non responders) as predicted by Model 1 (left panel) and by Model 2 (right panel). Each survey is represented by a circle. The black line is the identity line : if the satisfaction score was similar in responders and non responders, the circles would be close to the identity line. Circles below the identity lines mean that the mean satisfaction score in responders over-estimates the mean satisfaction score in all invited persons.


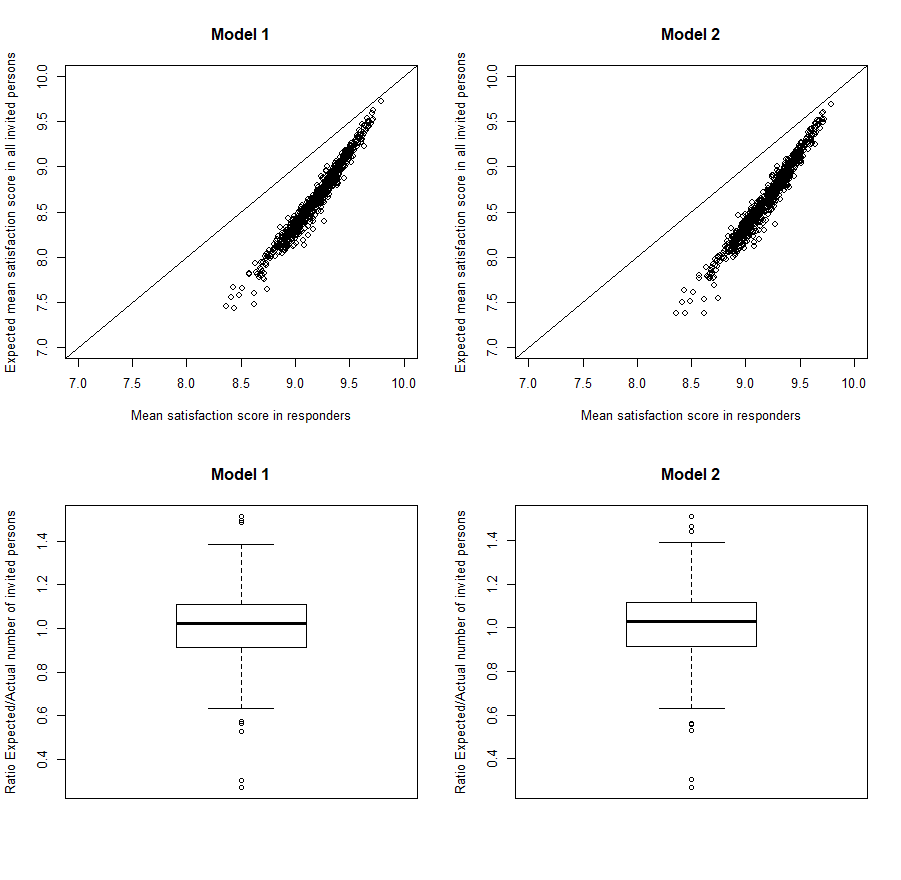

Supplement: Supplementary file 2 — Additional file 2. Estimation of the satisfaction-response function. [file 12913_2020_5012_MOESM2_ESM.docx]
